# A História Natural dos Tumores Cardíacos Primários em Crianças foi Modificada Recentemente?

**DOI:** 10.36660/abc.20230849

**Published:** 2024-03-13

**Authors:** Vera Demarchi Aiello

**Affiliations:** 1 Universidade de São Paulo Faculdade de Medicina Instituto do Coração do Hospital das Clínicas São Paulo SP Brasil Instituto do Coração do Hospital das Clínicas da Faculdade de Medicina da Universidade de São Paulo, São Paulo, SP – Brasil

**Keywords:** Criança, Feto/diagnóstico por imagem, Neoplasias Cardíacas/patologia, Esclerose Tuberosa/complicações, Coração Fetal/anormalidades

Nesta edição dos Arquivos Brasileiros de Cardiologia, Camargo et al.,^1^ apresentam sua experiência de 30 anos no diagnóstico de tumores cardíacos primários durante a vida fetal.^1^

Os autores focaram no grupo dos rabdomiomas, tumor cardíaco mais prevalente em fetos relatado na literatura^2^ e em sua casuística.

Conforme afirmam Camargo et al.,^1^ os rabdomiomas cardíacos podem sofrer regressão espontânea e somente quando causarem obstrução da via de saída ou arritmias que resistam ao tratamento médico a ressecção cirúrgica pode ser necessária. No entanto, uma abordagem terapêutica não invasiva foi recentemente adotada, com terapia materna com inibidores de mTOR e, se necessário devido ao crescimento persistente do tumor, tratamento pós-natal do paciente.^3^ Além disso, a maioria dos pacientes diagnosticados em vida fetal com rabdomiomas múltiplos também apresentam o complexo de esclerose tuberosa, e as manifestações sistêmicas - inclusive as neurológicas - devem ser convenientemente tratadas.

mTOR é uma quinase que integra vários sinais para controlar o crescimento e a tradução celular. O complexo da esclerose tuberosa deriva da presença de variantes de genes que codificam proteínas que, em condições normais, inibem a ativação da via mTOR. Mutações nestas proteínas podem resultar na ativação permanente do mTOR e na proliferação celular descontrolada.^4^

Sirolimus e everolimus são os medicamentos utilizados para inibir o crescimento de rabdomiomas, mas foram inicialmente utilizados como imunossupressores em transplantes de órgãos e também no tratamento de linfangioleiomiomatose em adultos (LAM). Foi relatada regressão completa dos rabdomiomas cardíacos após o uso desses inibidores de mTOR.^5 , 6^

No Brasil, o Sirolimus está hoje disponível para pacientes do Sistema Único de Saúde (SUS) após indicação clínica. O período de estudo de Camargo et al.,^1^ entretanto, foi de 30 anos e, para a maioria de seus pacientes, os medicamentos inibidores de mTOR não estavam disponíveis.

Considerando a ampla utilização dessas drogas em todo o mundo, pode-se considerar que uma história natural modificada dos rabdomiomas fetais ainda não foi descrita.

Outro procedimento considerado modificador de neoplasias cardíacas inoperáveis é o transplante cardíaco.^7^ Às vezes utilizado como opção para neoplasias recorrentes malignas ou não malignas, o transplante pode ser o procedimento de escolha para tumores localizados em topografias críticas. Também tem sido realizada em lactentes e crianças para tumores que ocupem grande parte das paredes ventriculares, como ocorre frequentemente com fibromas. Alguns relatos de crianças pequenas com fibromas cardíacos submetidas a transplante já foram publicados.^8^

Neonatos com hamartomas cardíacos mais raros se beneficiariam da mesma terapia, por exemplo, casos de hamartomas de cardiomiócitos maduros, que, embora não sejam infiltrativos, podem ocupar grandes áreas das paredes ventriculares.

## Morfologia das lesões nodulares do coração fetal e neonatal

As lesões nodulares mais frequentes do coração fetal e neonatal são os rabdomiomas e os fibromas, considerados hamartomas.

Nas células do rabdomioma, a falta de marcadores imuno-histoquímicos proliferativos indica que essas lesões são mais provavelmente hamartomas do que verdadeiras neoplasias.^9^

**Figura 1:**
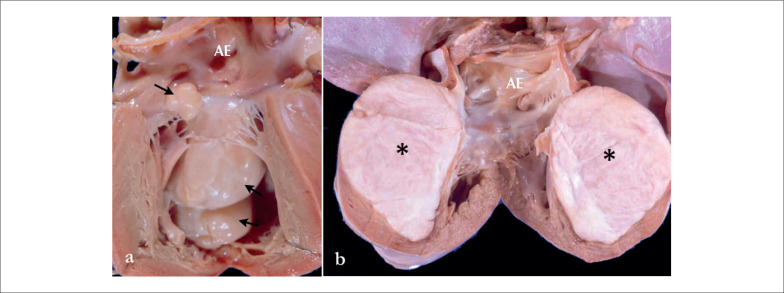
Aspectos macroscópicos dos tumores cardíacos primários mais frequentes em neonatos. O painel a) mostra um coração neonatal com múltiplos rabdomiomas no interior das câmaras cardíacas esquerdas, de diferentes tamanhos (setas). No painel b) observamos o coração de um neonato com uma enorme massa intramural (septal) esbranquiçada, substituindo parcialmente o miocárdio e também projetando-se para dentro da cavidade ventricular (asteriscos). AE: átrio esquerdo.

Embora os rabdomiomas sejam frequentemente lesões múltiplas e bem demarcadas, os fibromas são geralmente únicos e têm limites infiltrativos ( Figura 1 ).

**Figura 2:**
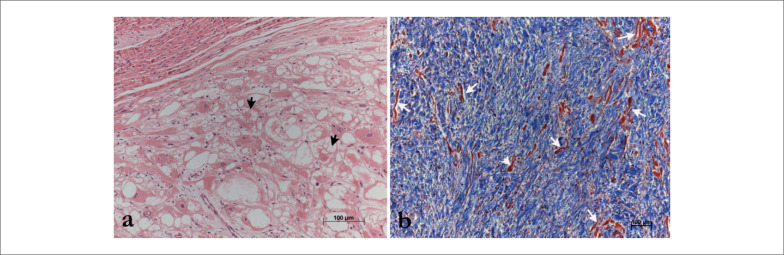
Características histológicas de rabdomioma e fibroma. Em a) um rabdomioma ventricular típico apresenta grandes células vacuolizadas, algumas com aparência de “células em aranha” (setas pretas). O painel b) mostra a periferia de um fibroma cardíaco, com células miocárdicas aprisionadas (setas brancas) entre o denso estroma de colágeno (azul). Coloração hematoxilina-eosina e tricrômico de Masson, respectivamente.

Histologicamente, rabdomiomas e fibromas também são bastante distintos. Os primeiros são compostos por células vacuoladas grandes e poligonais com limites claros e ocasionalmente finos filamentos de citoplasma que unem o núcleo à membrana celular, dando-lhes a aparência de “células em aranha” ( Figura 2 ).

Por outro lado, os fibromas apresentam células fusiformes bem orientadas (fibroblastos) imersas em um estroma colagênico denso, que aprisiona células miocárdicas normais na periferia da lesão ( Figura 2 ).

Em resumo, os avanços nos métodos diagnósticos e terapêuticos já alteraram o prognóstico de crianças nascidas com tumores cardíacos primários. A esperança é que haja mais avanços para que o tratamento da maioria dos tumores possa ser iniciado ainda durante a vida fetal.
