# Supplementary material for: A História Natural dos Tumores Cardíacos Primários em Crianças foi Modificada Recentemente?
Source: Arq Bras Cardiol. 2024 Mar 13;121(1):e20230849. [Article in Portuguese] doi: 10.36660/abc.20230849 (PMC11081200; doi:10.36660/abc.20230849)
Supplement: Translation

# Translation: Has the Natural History of Primary Heart Tumors in Children Been Modified Recently?

**Authors:** Vera Demarchi Aiello

**Affiliations:** 1 São Paulo SP Brazil Instituto do Coração do Hospital das Clínicas da Faculdade de Medicina da Universidade de São Paulo, São Paulo, SP – Brazil

**Keywords:** Child, Fetus/diagnosis imaging, Heart Neoplasms/pathology, Tuberous Sclerosis/complications, Fetal Heart/abnormalities

In this issue of Arquivos Brasileiros de Cardiologia, Camargo et al.^1^present their 30-year experience in the diagnosis of primary heart tumors during fetal life.^1^

The authors focused on the group of rhabdomyomas, the most prevalent heart tumor in fetuses reported in the literature^2^and in their casuistic.

As stated by Camargo et al.,^1^cardiac rhabdomyomas may undergo spontaneous regression, and only when causing outflow tract obstruction or arrhythmias that resist medical management, surgical resection may be necessary. However, a noninvasive therapeutic approach has been recently adopted, with maternal therapy with mTOR inhibitors and, if needed because of persistent tumor growth, postnatal treatment of the patient.^3^Moreover, most patients diagnosed in fetal life with multiple rhabdomyomas also have the tuberous sclerosis complex, and the systemic manifestations- including the neurologic ones- should be conveniently treated.

mTOR is a kinase that integrates various signals to control cellular growth and translation. Tuberous sclerosis complex derives from the presence of variants of genes that encode proteins that, in normal conditions, inhibit mTOR pathway activation. Mutations in these proteins can result in permanent mTOR activation and uncontrolled cell proliferation.^4^

Sirolimus and everolimus are the drugs used to inhibit the growth of rhabdomyomas but were initially used as an immunosuppressor in organ transplantation and also in the treatment of lymphangioleiomyomatosis in adults (LAM). Complete regression the heart rhabdomyomas has been reported after the use of this mTOR inhibitors.^5 , 6^

In Brazil, Sirolimus is nowadays available for patients from the public Unified Health System (SUS) after clinical indication. The period of study of Camargo et al.,^1^however, was 30 years, and for most of their patients, the mTOR inhibitor drugs were not available.

Considering the widespread utilization of these drugs all over the world, one can consider that a modified natural history of fetal rhabdomyomas is yet to be described.

Another procedure that is considered a modifier for inoperable heart neoplasms is heart transplantation.^7^Sometimes used as an option for malignant or non-malignant recurrent neoplasms, transplantation can be the procedure of choice for tumors located in critical topographies. It has also been performed in infants and children for tumors occupying a great part of the ventricular walls, as it occurs frequently with fibromas. Some reports of small children with heart fibromas submitted to transplant have already been published.^8^

Neonates with rarer heart hamartomas would benefit from the same therapy, for example, cases of hamartoma of mature cardiomyocytes, which, although not infiltrative, can occupy large areas of the ventricular walls.

## Morphology of the nodular lesions of the fetal and neonatal heart

The most frequent nodular lesions of the fetal and neonatal heart are rhabdomyomas and fibromas, which are considered hamartomas.

In rhabdomyoma cells, the lack of proliferative immunohistochemical markers indicates that these lesions are more likely hamartomas rather than true neoplasms.^9^

While rhabdomyomas are frequently multiple and well-demarcated lesions, fibromas are usually single and have infiltrative boundaries ( Figure 1 ).

**Figure 1:** Gross features of the most frequent primary heart tumors in neonates. Panel a) shows a neonatal heart with multiple rhabdomyomas inside the left cardiac chambers of different sizes (arrows). In panel b) we observe the heart from a neonate with a huge intra-mural (septal) whitish mass, partially replacing the myocardium and also protruding into the ventricular cavity (asterisks). LA: left atrium.

Histologically, rhabdomyomas and fibromas are also quite distinct. The first ones are composed of large and polygonal vacuolated cells with clear limits and occasionally fine strands of cytoplasm joining the nucleus to the cell membrane, giving them the appearance of “spider cells” ( Figure 2 ).

**Figure 2:** Histological features of rhabdomyoma and fibroma. In a) a typical ventricular rhabdomyoma shows large vacuolated cells, a few with the appearance of “spider cells” (black arrows). Panel b) shows the periphery of a cardiac fibroma, with entrapped myocardial cells (white arrows) among the dense collagen stroma (blue). Hematoxylin-eosin stain and Masson's trichrome stain, respectively.

On the other hand, fibromas show well-oriented spindle cells (fibroblasts) immersed in a dense collagenic stroma, which entraps normal myocardial cells at the periphery of the lesion ( Figure 2 ).

In summary, advances in diagnostic and therapeutic methods have already changed the prognosis of children born with primary cardiac tumors. The hope is that more advances come so that the treatment for most tumors can be started during fetal life.
